# Cervical Cancer Screening Barriers Among Citizens of Jeddah

**DOI:** 10.7759/cureus.50797

**Published:** 2023-12-19

**Authors:** Ahmed A Ghazi, Husain M Alturkistani, Anas M Alturkistani, Hamzah Y Alhajuj, Asseel A Alaidarous

**Affiliations:** 1 Obstetrics and Gynecology, Jeddah University, Jeddah, SAU; 2 Radiology, King Saud University, Riyadh, SAU; 3 Medicine, Jeddah University, Jeddah, SAU

**Keywords:** social barrier, screening test, pap smear, hpv, cervical cancer

## Abstract

Introduction: The cervix, a cylindrical structure made of stroma and epithelium, is the lowest point of the uterus. A tissue-proven biopsy of the cervix with histological confirmation is necessary for aberrant cytologic results of Papanicolaou (Pap) smears to rule out cervical cancer. This study investigates barriers to cervical cancer screening among women.

Methodology: A cross-sectional study including 665 Saudi Arabian women residing in Jeddah between the ages of 21 and 65 years was carried out from May to November 2023. A four-part online survey was used to gather the data, which included questions about demographics, cervical cancer screening status, screening barriers, and predictors of cervical cancer screening.

Results: The most common barrier to Pap test screening was "have not been to a doctor/no regular provider" (39.7%, N = 251), followed by "lack of provider recommendation/or limited or no information in the community" (30.4%, N = 192) and "low priority/did not perceive need/being healthy" (27.7%, N = 175).

Conclusion: The study provides valuable insights into the factors influencing cervical cancer screening in Jeddah, Saudi Arabia. The low prevalence of screening indicates a need for increased awareness and targeted interventions to improve uptake.

## Introduction

The cervix, a cylindrical structure made of stroma and epithelium, is the lowest point of the uterus. The ectocervix is lined with squamous epithelium and projects into the vagina. Columnar epithelium lines the endocervical canal, which connects the internal and external os [1]. Squamous cell carcinomas, which cause cervical cancer, can also affect the uterus, pelvis, lymph glands, and other bodily systems in severe cases. Cervical cancer and the human papillomavirus (HPV) are known to be associated. There are over 100 different forms of HPV identified, although not all of them are known to increase the risk of cervical cancer. HPV is a member of the virus family [2]. The human papillomavirus, or HPV, is considered to be the most prevalent sexually transmitted virus in the United States and one of the most common causes of sexually transmitted diseases (STDs) in both men and women globally [3]. It has been shown that the most complicated class of human pathogenic viruses is papillomaviruses [4]. HPVs can also be classified as high risk or low risk based on their relationship to precursor lesions and cervical cancer. The low-risk HPV types are 6, 11, 42, 43, and 44. HPV types 16, 18, 31, 33, 34, 35, 39, 45, 51, 52, 56, 58, 59, 66, 68, and 70 are among the high-risk varieties. Some HPV varieties that are more commonly detected in squamous intraepithelial lesions (SILs) but less frequently discovered in malignancies are included in the high-risk group [3]. Young age at first coitus, several sexual partners, high parity, and a history of other STDs are epidemiologic risk factors for the development of cervical cancer. About 15% of cervical cancers are adenocarcinomas, while 80% of tumors are squamous cell cancer [5]. Because cervical cancer has a lengthy pre-invasive phase, it is curable [6]. Reducing death rates from cervical cancer in women requires early identification and treatment. Screening for cervical cancer using a population-based Papanicolaou (Pap) smear or cytology is a crucial secondary preventative approach [6]. For the past 60 years, the cornerstone of cervical cancer screening has been the Pap smear or cervical cytology. Cytology has the benefits of being inexpensive and easy to use. A biopsy and histological confirmation are necessary for an aberrant cytologic result [6].

In 2017, a study conducted in the United States discovered that barriers to cervical cancer screening led to disparities in cervical cancer screening rates [7]. These constraints have been roughly classified as personal and structural impediments. Fear of discovering cancer, shame, a lack of knowledge of risk factors, screening by a male physician, recent immigration status, and the presence of chronic conditions are all personal barriers studied in the research [7]. Even when some of these structural barriers are addressed, such as through free screening programs, some low-income women do not take advantage of these chances. As a result, a greater knowledge of the hurdles and misconceptions about cervical cancer risk factors among this demographic is required [7]. Another study in Europe discovered that cervical cancer screening was efficient in reducing the incidence and mortality from the disease, prompting European governments to develop screening programs [8]. However, migrant women participate in screening at a lesser rate than citizens. In November 2019, electronic peer-reviewed resources were searched for research on factors related to migrants' participation in cervical cancer screening in European Union/European Free Trade Association countries, using broad search terms [8]. The most common barriers were a lack of information, a shortage of female healthcare practitioners, insufficient language abilities, and emotional reactions to the test (particularly fear, shame, and discomfort) [8].

A self-administered questionnaire was used in this cross-sectional study of 506 randomly chosen Saudi female secondary school teachers in Al Hassa, Saudi Arabia, to gauge their level of familiarity with risk factors and symptoms of cervical cancer in relation to perceived risk and to describe cervical cancer screening compliance [9]. A total of 65.4% and 63.4% of the female Saudi teachers who were included in the study were deemed to have less understanding of cervical cancer risk factors and early symptoms, respectively [9]. Risk factor awareness and increased cervical cancer awareness were strongly correlated with a high loading eigenvalue of 4.392 [9], which accounts for 30.8% of the barriers to use. Exploratory factor analysis revealed that personal worries (of screening being embarrassing) were the main obstacle to cervical cancer screening, followed by healthcare-related issues [9]. A cross-sectional study was conducted between May and November 2021 among 385 women aged 21-65 years who live in Jeddah, Saudi Arabia [10]. Demographic information, cervical cancer screening status, cervical cancer screening predictors, and screening barrier data were gathered using a four-part online survey. The factors that were discovered to be significantly associated with the screening status (having a Pap test) in the univariate analysis are growing age [10], education level, monthly income, perceived risk of developing cervical cancer, source of information regarding Pap tests, having a family doctor, recommendation to have a Pap tests by the family doctor, having a gynecological examination, visiting a gynecologist in the past, history of prior gynecological complaint, and history of abortion [10]. Only four variables were shown to be substantially linked with the screening status in the multivariable analysis, including age, monthly income, having previously undergone a gynecological examination, and the recommendation by the family physician, which by far had the most impact [10].

## Materials and methods

This cross-sectional study set out to evaluate the cervical cancer screening status, predictors, and obstacles among female Jeddah residents aged 21 to 65 years who had previously participated in sexual activity at some point in their lives. The research was carried out between August and November of 2023, with a target sample size of 385 women who satisfied the predetermined inclusion requirements.

Female residents of Jeddah between the ages of 21 and 65 years who have engaged in sexual activity at some point in their lives and who volunteered to participate in the study were included. Those who did not meet the inclusion criteria (females younger than 21 years or older than 65 years, those who are due, or who have never engaged in sexual activity) were excluded [10].

Sample size

With a 50% cervical cancer screening assumption, a 5% margin of error, and a 95% confidence level, Raosoft's equation was used to compute the sample size, which came out to be 385. Because of the epidemic, snowball sampling was used, and the poll was made available online. Having a positive reaction after a Pap test indicates participation in cervical cancer screening. We discussed awareness of cervical cancer, perceived risk, knowledge, and opinions regarding possibilities. We examined knowledge of Pap tests, sources of information, the existence of a family doctor, the doctor's gender, suggestions, gynecological history, frequency of visits, exams, number of children, history of abortions, and, if relevant, the reasons for not getting a Pap test.

Sampling method

To acquire data, a Google Forms (Google, Mountain View, CA) anonymous survey was used. The survey was divided into two sections: the first collected personal information (age, education, and socioeconomic position), and the second evaluated the prevalence, predictors, and obstacles associated with cervical cancer screening. A study permission form detailing the purpose of the research, the demographic it is intended for, and the guarantee of anonymity was provided on the first page of the survey. The option to volunteer ("yes" or "no") was presented to participants. Only those who answered "yes" moved on to the survey. Demographics, cervical cancer screening, predictors, and obstacles were all addressed in the questionnaire. Demographics such as age, marital status, nationality, place of residence in Saudi Arabia, education, employment, and income were among the demographic details provided.

Data analysis

Using univariate binomial regression, each variable's relationship to the outcome (having had cervical cancer screening) was evaluated. A multivariate binary logistic regression model was constructed using variables that showed a significant (p < 0.05) connection (or near significant association; p < 0.1) with the outcome (screening status) in the univariate studies. Bivariate Pearson correlation was used to evaluate the multivariate binary regression model's premise. A significance level of p < 0.05 was used for every analysis.

Ethical considerations

Under application number UJ-REC-156, the study was approved by the University of Jeddah's Institutional Review Board (IRB). At the start of the survey, each participant gave their informed consent.

## Results

**Figure 1:**
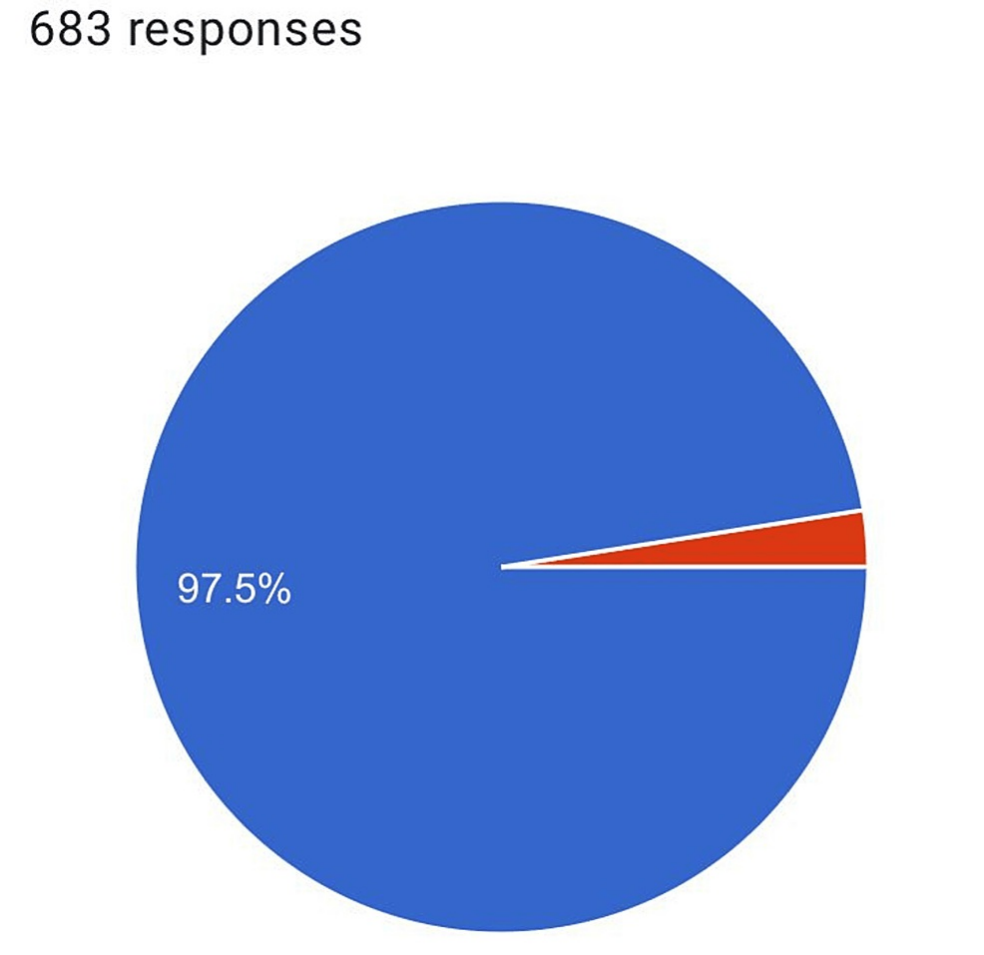
The number of acceptance to participate

Out of 683 responses, 97.5% (N = 665) accepted to participate and only 2.5% (N = 18) did not accept (Figure 1).

The results showed that out of 665 women surveyed, their ages ranged from 16 to 70 years, with a mean age of 35.33 years. A total of 39.4% (N = 262) were between 25 and 45 years old, 35.3% (N = 235) were under 25 years old, and 25.3% (N = 168) were over 45 years old (Table 1). We found that 178 out of 665 (26.8%, N = 178) had a Pap test in the past, nearly half of them were in the past three years (14.3%, N = 95), and the other 485 out of 665 (73.2%, N = 485) never had a Pap test (Table 2). The majority (93.1%, N = 619) were Saudi nationals, while 6.9% (N = 46) were non-Saudis (Table 1). In terms of marital status, approximately half (51.6%, N = 343) were married, 38.5% (N = 256) were single, 4.4% (N = 29) were divorced, and 5.6% (N = 37) were widowed (Table 1). Concerning their residence in Jeddah, 52.2% (N = 347) lived in the north, 18.9% (N = 126) lived in the south, 17.6% (N = 117) lived in the middle, and 11.3% (N = 75) lived in the east (Table 1). Education-wise, the majority (67.4%, N = 448) held a bachelor's degree, 17.3% (N = 115) had school-level education (primary, middle, or high school), 7.7% (N = 51) had a diploma, and 7.7% (N = 51) had a master's degree. Regarding monthly income, 51.4% (N = 342) earned 4000 Saudi riyals (SR) and above, while 48.6% (N = 323) earned less than 4000 SR (Table 1). We found that 26.8% (N = 178) of the total 665 had previously undergone a Pap test, while 73.2% (N = 485) had not. Additionally, 14.3% (N = 95) of the participants had undergone a Pap test within the last three years (Table 2).

**Table 1:** Demographic characteristics SR: Saudi riyal.

Variables	Categories	N	%
Age range: 16 to 70, mean = 35.33 ± 12.99	Less than 25 years	235	35.3
	From 25 to 45 years	262	39.4
	More than 45 years	168	25.3
Nationality	Saudi	619	93.1
	Non-Saudi	46	6.9
Marital status	Single	256	38.5
	Married	343	51.6
	Divorced	29	4.4
	Widowed	37	5.6
Residence	North	347	52.2
	South	126	18.9
	Middle	117	17.6
	East	75	11.3
Education	School (primary, middle, or high school)	115	17.3
	Diploma	51	7.7
	Bachelor	448	67.4
	Master	51	7.7
Income	Less than 4000 SR	323	48.6
	4000 SR and more	342	51.4

**Table 2:** Screening status

Questions	N	%
Have you ever had a Pap test in the past?		
Yes	178	26.8
No	485	73.2
Have you had a Pap test in the past three years?		
Yes	95	14.3
No	568	85.7

The results revealed that 84.3% (N = 559) of the respondents had heard about a disease called cervical cancer. Among them, 47.2% (N = 314) believed they are much below average to had a chance of developing cervical cancer compared to other women of their age, with 25.4% (N = 169) considering it below average, 23.3% (N = 155) considering it average, 2.4% (N = 16) considering above average, and 1.7% (N = 11) considering much above average. Additionally, 39.4% (N = 262) of the participants were aware of cervical cancer screening (Pap test). Of those, 22.6% (N = 150) learned about it through social media/television, 15% (N = 100) from healthcare providers/doctors, 8.1% (N = 54) from relatives or friends, and 2.7% (N = 18) from brochures/posters.

Furthermore, among the respondents, 24.4% (N = 162) reported having a family doctor, with the majority (70%, N = 112) being female and 30% (N = 47) male. Only 43% of the participant's family doctor (N = 70) recommended a Pap test to them. In terms of medical history, 31.6% (N = 210) had previous gynecological problems (such as abnormal bleeding or vaginal discharge), 51.4% (N = 342) had undergone previous gynecological examinations, and 62.9% (N = 418) had previously visited a gynecologist. Moreover, 27.2% (N = 181) had a history of previous abortions, while the majority (42.9%, N = 285) did not have any children, 39.6% (N = 263) had from one to four children, and 17.5% (N = 116) had more than four children (Table 3).

**Table 3:** Predictors for Pap test screening

Questions	N	%
Have you heard about a disease called cervical cancer?		
Yes	559	84.3
No	104	15.7
Compared to other women of your age, what do you think your chances of getting cervical cancer are?		
Much below average	314	47.2
Below average	169	25.4
Average	155	23.3
Above average	16	2.4
Much above average	11	1.7
Have you ever heard of cervical cancer screening (Pap test)?		
Yes	262	39.4
No	403	60.6
How did you learn about cervical cancer screening (Pap test)?		
Healthcare provider/doctor	100	15
Brochures/posters	18	2.7
Social media/television	150	22.6
Relative or friends	54	8.1
I have not heard about the Pap test	343	51.6
Do you have a family doctor (or regularly visit a primary healthcare center)?		
Yes	162	24.4
No	503	75.6
Family doctor's gender		
Male	47	7.1
Female	112	16.8
I don't have a family doctor	506	76.1
Has your family doctor ever recommended a Pap test?		
Yes	70	10.5
No	595	89.5
Previous gynecological problem (abnormal bleeding or vaginal discharge, or others)		
Yes	210	31.6
No	455	68.4
Previous gynecological examination		
Yes	342	51.4
No	323	48.6
Previous visit to a gynecologist		
Yes	418	62.9
No	247	37.1
Number of children		
No children	285	42.9
From 1 to 4	263	39.6
More than 4	116	17.5
Previous abortion		
Yes	181	27.2
No	484	72.8

The results showed the most common barriers to Pap test screening were "have not been to a doctor/no regular provider" (39.7%, N = 251), "lack of provider recommendation/or limited or no information in the community" (30.4%, N = 192), "low priority/did not perceive need/being healthy" (27.7%, N = 175), "do not have time" (18.7%, N = 118), "fear of the screening test results" (14.7%, N = 93), "the test is embarrassing" (12.8%, N = 81), "cost too much or no health insurance" (10.3%, N = 65), "negative experience with previous examination or procedure" (9.2%, N = 58), and "could not find appointment" (3.5%, N = 22). There were only three (0.5%) who had no specific reason (Table 4). So we can say that the most common barriers among those participants for Pap test screening were "have not been to a doctor/no regular provider," followed by "lack of provider recommendation/or limited or no information in the community" and "low priority/did not perceive need/being healthy."

**Table 4:** Barriers to Pap test screening

No.	Barriers	N	%
1	Have not been to a doctor/no regular provider	251	39.7
2	Lack of provider recommendations/or limited or no information in the community	192	30.4
3	Cost too much or no health insurance	65	10.3
4	Do not have time	118	18.7
5	Could not find an appointment	22	3.5
6	Low priority/did not perceive need/being healthy	175	27.7
7	Fear of the test or negative experience with previous examination or procedure	58	9.2
8	The test is embarrassing/or lack of female screener	81	12.8
9	Fear of the screening test results	93	14.7
10	Do not know a specific reason	3	0.5

This table showed that women whose family doctors have recommended a Pap test were about three times more likely to have undergone the test compared to those without recommendations (odds ratio = 3.057, P-value = 0.014). Women with previous gynecological issues are about 3.3 times more likely to have had a Pap test compared to those without such problems (odds ratio = 3.257, P-value < 0.001). Women who have had previous gynecological examinations were about 9.4 times more likely to have undergone a Pap test compared to those who had no previous gynecological examinations (odds ratio = 9.406, P-value < 0.001). Women who had previous abortions were about 0.491 less likely to have undergone the test compared to those who had no previous abortions (odds ratio = 0.491, P-value = 0.008). Women who have learned about cervical cancer screening (Pap test) from a healthcare provider/doctor were about six times more likely to have undergone a Pap test compared to those who had not heard about Pap test (odds ratio = 6.051, P-value = 0.002). Women who had no children were about 0.062 less likely to have undergone the test compared to those who had more than four children (odds ratio = 0.062, P-value < 0.001) (Table 5).

**Table 5:** The predictors for Pap test screening in the past

No.	Variables	B	Sig.	Odds ratio
1	Have you heard about a disease called cervical cancer?	0.602	0.188	1.827
2	Have you ever heard of cervical cancer screening (Pap test)?	-0.099	0.819	0.906
3	Do you have a family doctor (or regularly visit a primary healthcare center)?	0.545	0.238	1.725
4	Has your family doctor ever recommended a Pap test?	1.117	0.014	3.057
5	Previous gynecological problem (abnormal bleeding or vaginal discharge, or others)	1.181	<0.001	3.257
6	Previous gynecological examination	2.241	<0.001	9.406
7	Previous visit to a gynecologist	-0.064	0.901	0.938
8	Previous abortion	-0.711	0.008*	0.491
Compared to other women of your age, what do you think your chances of getting cervical cancer are? (much below average)				
9	Below average	0.051	0.873	1.052
10	Average	0.06	0.848	1.061
11	Above average	0.355	0.687	1.426
12	Much above average	1.536	0.083	4.648
How did you learn about cervical cancer screening (Pap test)? (I have not heard about the Pap test)				
13	Healthcare provider/doctor	1.8	0.002	6.051
14	Brochures/posters	-0.644	0.476	0.525
15	Social media/television	-0.283	0.536	0.754
16	Relative or friends	0.113	0.837	1.12
Family doctor gender (I don't have a family doctor)				
17	Male	-1.111	0.099	0.329
18	Female	-0.12	0.805	0.887
Number of children (more than 4 children)				
19	No children	-2.786	<0.001	0.062
20	From 1 to 4 children	0.426	0.152	1.531

The analysis demonstrated a significant relationship between age and those who had undergone a Pap test (138.873, P-value < 0.001). The highest percentage of screenings occurred among individuals aged over 45 years. Additionally, for individuals who had been screened within the past three years (138.873, P-value < 0.001), the highest percentage was observed among those aged between 25 and 45 years.

Moreover, the data revealed a significant association between marital status and Pap test screenings (170.013, P-value < 0.001), with the highest percentage of screenings observed among married women. This trend was similarly observed for those who had been screened within the past three years (65.011, P-value < 0.001).

Residence in Jeddah showed a significant association with those who had undergone a Pap test (15.971, P-value < 0.001), particularly in the northern region. However, there was no significant association with those who had undergone screenings in the past three years.

Additionally, education level exhibited a significant relationship with both the individuals who had ever undergone a Pap test (49.639, P-value < 0.001) and those who had been screened within the past three years (24.975, P-value < 0.001). The highest percentage of screenings was observed among individuals with a bachelor's degree.

Similarly, monthly income was significantly associated with those who had undergone a Pap test (44.27, P-value < 0.001) and those who had been screened within the past three years (12.587, P-value < 0.001). The highest percentage of screenings occurred among those with a monthly income of 4000 SR or more.

However, there was no significant association found with nationality (Table 6).

**Table 6:** The association between screening and demographic characteristics SR: Saudi riyal.

Variables	Categories	Pap test	
		In the past	In the past 3 years
Age	Less than 25 years	5	5
		2.80%	5.30%
	From 25 to 45 years	82	56
		46.10%	58.90%
	More than 45 years	91	34
		51.10%	35.80%
	X^2^	138.873	43.544
	P-value	<0.001	<0.001
Nationality	Saudi	165	88
		92.70%	92.60%
	Non-Saudi	13	7
		7.30%	7.40%
	X^2^	0.050	0.032
	P-value	0.832	0.858
Marital status	Single	4	3
		2.20%	3.20%
	Married	134	84
		75.30%	88.40%
	Divorced	9	3
		5.10%	3.20%
	Widowed	31	5
		17.40%	5.30%
	X^2^	170.013	65.011
	P-value	<0.001	<0.001
Residence in Jeddah	North	111	49
		62.40%	51.60%
	South	24	15
		13.50%	15.80%
	Middle	33	25
		18.50%	26.30%
	East	10	6
		5.60%	6.30%
	X^2^	15.971	7.777
	P-value	0.001	0.051
Education	Diploma	14	8
		7.90%	8.40%
	Bachelor	86	51
		48.30%	53.70%
	School (primary, middle, or high school)	50	17
		28.10%	17.90%
	Master	28	19
		15.70%	20.00%
	X^2^	49.639	24.975
	P-value	<0.001	<0.001
Income	Less than 4000 SR	48	30
		27.00%	31.60%
	4000 SR and more	130	65
		73.00%	68.40%
	X^2^	44.827	12.587
	P-value	<0.001	<0.001

There was a significant association between women who had undergone a Pap test and age, marital status, residence in Jeddah, education, and income, but no significant association with nationality.

## Discussion

The study aimed to assess the cervical cancer screening status, predictors, and barriers among women aged 21-65 years in Jeddah, Saudi Arabia. The findings provide valuable insights into the factors influencing cervical cancer screening uptake.

This study found only 33.4% of women in Jeddah had undergone a Pap smear in comparison to another study done in 2017 in Al Hassa, Saudi Arabia, with only 17.2% of 506 participants [9]. Another study conducted in Turkey in 2014 found that only 1.1% of 800 participants did take a Pap test [11]. Even though our participants are more likely to undergo Pap tests, it still raises concerns about the overall low adherence to cervical cancer screening guidelines. This calls for urgent attention to understanding the root causes of this underutilization of screening services and developing targeted interventions to increase awareness and uptake.

The multivariable analysis highlighted age, monthly income, previous gynecological examination, and family doctor recommendation as the most influential predictors of Pap smear uptake. In contrast to our study, a study conducted in 2017 in Al Hassa, Saudi Arabia stated that living in an urban area is the most common predictor to be screened by Pap test, and not age or knowledge [9]. The dominance of these factors suggests that both socioeconomic and healthcare provider-related variables significantly impact screening behavior. It emphasizes the need for healthcare professionals to play a more proactive role in recommending and educating women about the importance of cervical cancer screening.

Also, the observed associations between demographic characteristics and screening uptake provide valuable insights for designing targeted interventions. The findings highlight the importance of considering age, marital status, residence, education, and income levels when tailoring awareness campaigns. Strategies should be designed to reach specific demographic groups that are less likely to undergo screening.

Furthermore, the identification of barriers, such as a lack of regular doctor visits, absence of provider recommendations, and low-priority needs, sheds light on specific challenges that need to be addressed. The study conducted in 2017 in Al Hassa, Saudi Arabia found that the barriers fit into three major categories: personal, health care, and culture-related barriers factor. The personal factors in their study were fear of results, and being shy from the test itself; on the other hand, healthcare-related factors that we found in our study as a barrier were no sites for screening, decreased knowledge about cervical cancer, and lack of education about cervical cancer [9]. Strategies to improve healthcare access, increase healthcare provider recommendations, and enhance the perceived importance of screening could be crucial in overcoming these barriers.

The study also highlights the important role of family doctors in influencing women's decisions to undergo cervical cancer screening. A study conducted in 2020 in Chile and Los Angeles used a trial of two methods to convince women to take a Pap smear, one by evidence-based film and another by storytelling film. They found the participants who watched the storytelling film reserved for a Pap test way more often than the evidence-based film [12]. The fact that only 10.2% of family doctors had recommended a Pap test suggests a significant opportunity for improving communication between healthcare providers and patients. Training programs and guidelines for healthcare professionals could be implemented to ensure they actively recommend and discuss screening options with eligible patients.

There is a need for education and awareness campaigns. Social media and healthcare providers emerged as significant sources of information about cervical cancer screening. Strengthening these channels, along with targeted outreach to specific demographic groups identified in the study, could improve overall awareness and knowledge.

Limitations

One limitation of the study is its cross-sectional design. Cross-sectional studies capture data at a specific point in time, making it challenging to establish causal relationships. Factors influencing cervical cancer screening behavior may change over time. Future research employing longitudinal designs could provide a more sustained understanding of the dynamic nature of these factors.

Also, the reliance on self-reported data for variables such as screening history, awareness, and demographic information introduces the potential for recall bias. Respondents may inaccurately recall or overstate their screening history, leading to an underestimation or overestimation of the true prevalence of cervical cancer screening.

The study was conducted in Jeddah, Saudi Arabia, and may not fully represent the diverse socio-cultural contexts within the country. Generalizing the findings to the broader Saudi population or other cultural contexts should be done cautiously. Regional variations in healthcare accessibility, cultural attitudes, and socioeconomic factors may influence cervical cancer screening differently in various parts of the country.

## Conclusions

The study provides valuable insights into the factors influencing cervical cancer screening in Jeddah, Saudi Arabia. In this study, out of 665 participants, only 26.8% (N = 178) had a Pap test in the past and the rest (73.2%, N = 485) did not. This low prevalence of screening indicates a need for increased awareness and targeted interventions to improve uptake. Healthcare professionals, especially family doctors, play a pivotal role in influencing women's decisions to undergo screening. Tailored awareness campaigns, particularly through healthcare providers and social media, can effectively address knowledge gaps and overcome barriers. This study was done in 2023 and the last study on the same topic was conducted two years ago. The major barriers were almost the same, i.e., insufficient perceived need because one is well, absence of advice from providers, and unavailability of test-related information.

## References

[1] Bhatla N, Aoki D, Sharma DN, Sankaranarayanan R (2021). Cancer of the cervix uteri: 2021 update. Int J Gynaecol Obstet.

[2] Bedford S (2009). Cervical cancer: physiology, risk factors, vaccination and treatment. Br J Nurs.

[3] Burd EM (2003). Human papillomavirus and cervical cancer. Clin Microbiol Rev.

[4] Zur Hausen H (1999). Papillomaviruses in human cancers. Proc Assoc Am Physicians.

[5] Moore DH (2006). Cervical cancer. Obstet Gynecol.

[6] Taneja N, Chawla B, Awasthi AA, Shrivastav KD, Jaggi VK, Janardhanan R (2021). Knowledge, attitude, and practice on cervical cancer and screening among women in India: a review. Cancer Control.

[7] Akinlotan M, Bolin JN, Helduser J, Ojinnaka C, Lichorad A, McClellan D (2017). Cervical cancer screening barriers and risk factor knowledge among uninsured women. J Community Health.

[8] Marques P, Nunes M, Antunes MD, Heleno B, Dias S (2020). Factors associated with cervical cancer screening participation among migrant women in Europe: a scoping review. Int J Equity Health.

[9] Salem MR, Amin TT, Alhulaybi AA, Althafar AS, Abdelhai RA (2017). Perceived risk of cervical cancer and barriers to screening among secondary school female teachers in Al Hassa, Saudi Arabia. Asian Pac J Cancer Prev.

[10] Alsalmi SF, Othman SS (2022). Cervical cancer screening uptake and predictors among women in Jeddah, Saudi Arabia. Cureus.

[11] Koç Z (2015). University students' knowledge and attitudes regarding cervical cancer, human papillomavirus, and human papillomavirus vaccines in Turkey. J Am Coll Health.

[12] Baezconde-Garbanati L, Agurto I, Gravitt PE (2019). Barriers and innovative interventions for early detection of cervical cancer. Salud Publica Mex.

